# When age means safety: Data to assess trends and differences on rule knowledge, risk perception, aberrant and positive road behaviors, and traffic crashes of cyclists

**DOI:** 10.1016/j.dib.2018.12.066

**Published:** 2018-12-23

**Authors:** Sergio A. Useche, Francisco Alonso, Luis Montoro, José M. Tomas

**Affiliations:** a DATS (Development and Advising in Traffic Safety) Research Group, INTRAS (Research Institute on Traffic and Road Safety), University of Valencia, Spain; b FACTHUM.Lab (Human Factor and Road Safety) Research Group, INTRAS (Research Institute on Traffic and Road Safety), University of Valencia, Spain; c Department of Methodology for the Behavioral Sciences, Faculty of Psychology, University of Valencia, Spain

## Abstract

This data article examines the association between age, knowledge of traffic rules, risk perception, risky and positive behaviors on the road and traffic safety outcomes of cyclists. The data was collected using a structured self-administrable and online-based questionnaire, applied to a full sample of 1064 cyclists. The data contains 4 parts: descriptive statistics; graphical trends for each study variable according to age; Post-Hoc (Tukey-HSD) comparisons between cyclists classified in the different age groups; and, finally, the dataset for further explorations in this regard. For further information, it is convenient to read the full article entitled “*Explaining Self-Reported Traffic Crashes of Cyclists: An Empirical Study based on Age and Road Risky Behaviors*” (Useche et al., 2019) [1].

## Specifications table

**Table:** 

Subject area	*Psychology*
More specific subject area	*Road safety and health behavior, sustainable transport modes, traffic-crash prevention, vulnerable road users*
Type of data	*Tables, graph, database*
How data was acquired	*Original data was collected through an international web-based survey. The data was consolidated and analyzed through the statistical software package IBM SPSS (version 23.0) for descriptive procedures and IBM SPSS AMOS (version 22.0) for structural/inferential ones*
Data format	*Filtered and analyzed*
Experimental factors	*Population consisted of a sample of regular cyclists, about which their (protective and risky) road behaviors, perception of road risks and self-reported level of knowledge about traffic regulations were analyzed*
Experimental features	*Study of age-based differences on cycling-related factors and road behaviors of cyclists through HSD (Post-Hoc) and graphical analyses*
Data source location	*Europe, Latin and North America*
Data accessibility	*Data is with this article*
Related research article	*Useche S, Alonso F, Montoro L., & Esteban, C. Explaining Self-Reported Traffic Crashes of Cyclists: An Empirical Study based on Age and Road Risky Behaviors. Saf Sci. 2018 113, 105–114**[1]*.

## Value of the data

• This data provides information on the profile (age, cycling patterns, psychosocial and behavioral issues, and traffic crashes) of a sample of 1064 cyclists of different countries.
• The risky and positive cycling behaviors of cyclists can be compared according to different user-related features, such as their age, gender, educative level and occupation, variables also contained in the annex dataset.
• The data could be compared with other samples/studies using the Behavioral-Questionnaire (BQ) approach to examine associations and trends on traffic behaviors and crashes among cyclists.
• This data can be used by other researchers and road safety practitioners to identify riskier patterns and propose evidence-based interventions grounded on the data provided by this large international sample of bicycle users.

## 1 Data

**Fig. 1:**
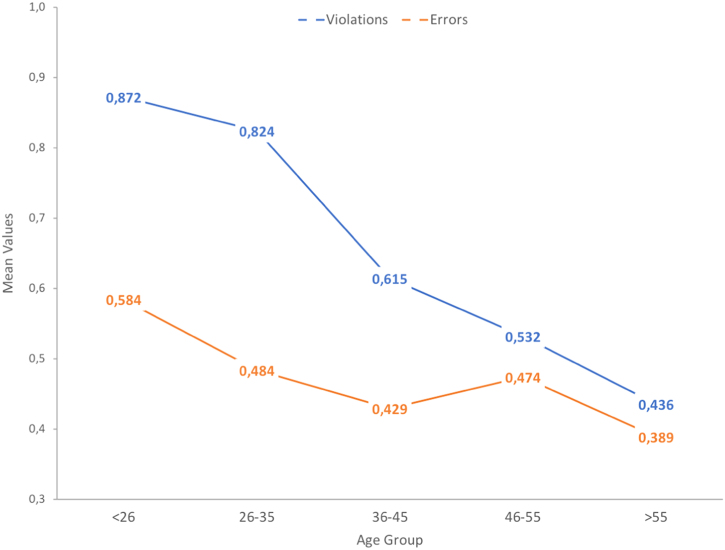
Trends in risky cycling behaviors (errors and violations) of cyclists according to their age group.

**Fig. 2:**
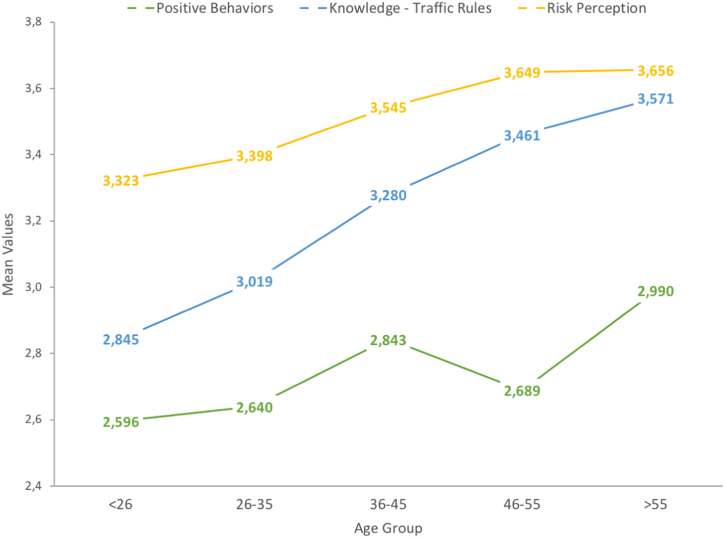
Trends on rule knowledge, risk perception and positive cycling behaviors according to age group.

**Fig. 3:**
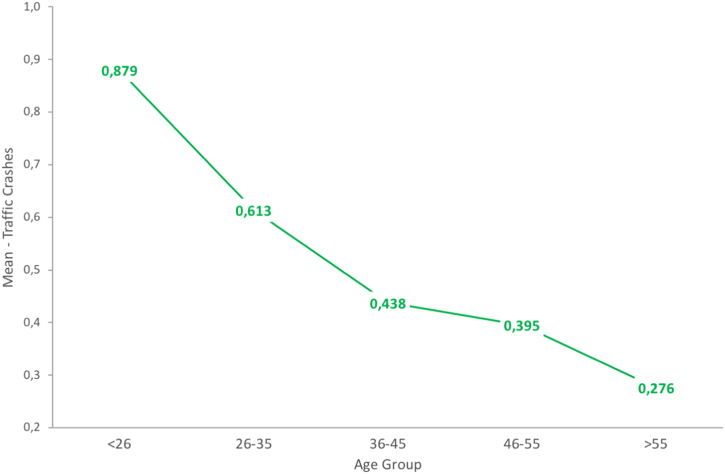
Trends on rule knowledge, risk perception and positive behaviors of cyclists according to their age group.

The dataset of this article provides information on a set of demographics, behavioral and crash-related factors of the sample, entirely composed of active cyclists. Table 1 shows the descriptive statistics obtained for all study variables included in this data article. Fig. 1 shows graphically the trends on self-reported aberrant cycling behaviors according to the age of cyclists, and Table 2 allows to identify the specific differences between cyclists of all age groups through a Post-Hoc analysis. In the same sense, Fig. 2 addresses protective factors: positive cycling behaviors, rule knowledge and risk perception, and Table 3 summarizes the statistical differences between age groups for these three variables. Finally, Fig. 3 shows the actual trends on traffic crashes suffered by cyclists of the different age groups during the last 5 years, and Table 4 presents the Post-Hoc-based significant differences found in traffic crash rates.

**Table 1:** Descriptive statistics of the variables contained in the data set.

**Study variable**	**Age group**											
	**Total**		**< 26**		**26–35**		**36–45**		**46–55**		**> 55**	
	**Mean**	**SD**	**Mean**	**SD**	**Mean**	**SD**	**Mean**	**SD**	**Mean**	**SD**	**Mean**	**SD**
Violations	0.749	0.46	0.872	0.44	0.824	0.49	0.615	0.39	0.532	0.34	0.436	0.33
Errors	0.505	0.50	0.584	0.38	0.484	0.41	0.429	0.36	0.474	0.38	0.389	0.31
Risky Behaviors	1.254	0.73	1.457	0.70	1.308	0.79	1.044	0.65	1.006	0.60	0.825	0.54
Positive Behaviors	2.684	0.62	2.596	0.55	2.640	0.63	2.843	0.64	2.689	0.69	2.990	0.58
Knowledge of Traffic Rules	3.083	0.71	2.845	0.72	3.019	0.69	3.280	0.62	3.461	0.52	3.571	0.47
Risk Perception	3.493	0.50	3.323	0.49	3.398	0.52	3.545	0.46	3.649	0.47	3.656	0.36
Traffic Crashes (5 years)	0.640	0.98	0.879	1.17	0.613	0.89	0.438	0.72	0.395	0.86	0.276	0.58

**Table 2:** Post-Hoc (Tukey HSD) analysis – mean comparisons for risky cycling behaviors of cyclists. Factor: age group.

**Dependent variable**	**(I) Age group**	**(J) Age group**	**Mean Diff. (I-J)**	**Std. Error**	**Sig.**		**95% CI**	
					***p***	**Level**	**Lower**	**Upper**
**Violations**	< 26	36–45	0.26	0.041	0.000	***	0.146	0.369
		46–55	0.340	0.046	0.000	***	0.215	0.465
		> 55	0.437	0.055	0.000	***	0.287	0.586
	26–35	36–45	0.209	0.042	0.000	***	0.094	0.325
		46–55	0.292	0.047	0.000	***	0.164	0.420
		> 55	0.388	0.056	0.000	***	0.236	0.541
	36–45	< 26	-0.257	0.041	0.000	***	-0.369	-0.146
		26–35	-0.209	0.042	0.000	***	-0.325	-0.094
		> 55	0.179	0.061	0.027	*	0.013	0.345
	46–55	< 26	-0.340	0.046	0.000	***	-0.465	-0.215
		26–35	-0.292	0.047	0.000	***	-0.420	-0.164
	> 55	< 26	-0.437	0.055	0.000	***	-0.586	-0.287
		26–35	-0.388	0.056	0.000	***	-0.541	-0.236
		36–45	-0.179	0.061	0.027	*	-0.345	-0.013
**Errors**	< 26	26–35	0.100	0.029	0.005	**	0.021	0.180
		36–45	0.155	0.036	0.000	***	0.056	0.254
		> 55	0.195	0.048	0.001	**	0.063	0.327
	26–35	< 26	-0.100	0.029	0.005	**	-0.180	-0.021
	36–45	< 26	-0.155	0.036	0.000	***	-0.254	-0.056
	> 55	< 26	-0.195	0.048	0.001	**	-0.327	-0.063
**Risky Behaviors**	< 26	26–35	0.148	0.053	0.044	*	0.003	0.294
		36–45	0.412	0.066	0.000	***	0.231	0.594
		46–55	0.450	0.074	0.000	***	0.248	0.652
		> 55	0.632	0.089	0.000	***	0.390	0.874
	26–35	< 26	-0.148	0.053	0.044	*	-0.294	-0.003
		36–45	0.264	0.068	0.001	**	0.077	0.451
		46–55	0.302	0.076	0.001	**	0.094	0.509
		> 55	0.483	0.090	0.000	***	0.237	0.730
	36–45	< 26	-0.412	0.066	0.000	***	-0.594	-0.231
		26–35	-0.264	0.068	0.001	**	-0.451	-0.077
	46–55	< 26	-0.450	0.074	0.000	***	-0.652	-0.248
		26–35	-0.302	0.076	0.001	**	-0.509	-0.094
	> 55	< 26	-0.632	0.089	0.000	***	-0.874	-0.390
		26–35	-0.483	0.090	0.000	***	-0.730	-0.237

*Notes:* *The mean difference is significant at the 0.05 level. **The mean difference is significant at the 0.01 level. ***The mean difference is significant at the 0.001 level.

**Table 3:** Post-Hoc (Tukey HSD) analysis – mean comparisons for positive behaviors, knowledge of traffic rules and risk perception of cyclists. Factor: Age group.

**Dependent variable**	**(I) Age group**	**(J) Age group**	**Mean Diff. (I-J)**	**Std. Error**	**Sig.**		**95% CI**	
					***p***	**Level**	**Lower**	**Upper**
**Positive Behaviors**	< 26	36–45	-0.248	0.057	0.000	***	-0.404	-0.091
		> 55	-0.395	0.076	0.000	***	-0.604	-0.186
	26–35	36–45	-0.204	0.059	0.005	**	-0.365	-0.042
		> 55	-0.350	0.078	0.000	***	-0.563	-0.138
	36–45	< 26	0.248	0.057	0.000	***	0.091	0.404
		26–35	0.204	0.059	0.005	**	0.042	0.365
	46–55	> 55	-0.301	0.090	0.007	**	-0.546	-0.056
	> 55	< 26	0.395	0.076	0.000	***	0.186	0.604
		26–35	0.350	0.078	0.000	***	0.138	0.563
		46–55	0.301	0.090	0.007	**	0.056	0.546
**Knowledge of Traffic Rules**	< 26	26–35	-0.174	0.050	0.005	**	-0.312	-0.037
		36–45	-0.435	0.062	0.000	***	-0.606	-0.265
		46–55	-0.616	0.070	0.000	***	-0.806	-0.426
		> 55	-0.726	0.083	0.000	***	-0.954	-0.499
	26–35	< 26	0.174	0.050	0.005	**	0.037	0.312
		36–45	-0.261	0.064	0.001	**	-0.437	-0.085
		46–55	-0.442	0.072	0.000	***	-0.637	-0.246
		> 55	-0.552	0.085	0.000	***	-0.784	-0.320
	36–45	< 26	0.435	0.062	0.000	***	0.265	0.606
		26–35	0.261	0.064	0.001	**	0.085	0.437
		> 55	-0.291	0.093	0.015	*	-0.544	-0.038
	46–55	< 26	0.616	0.070	0.000	***	0.426	0.806
		26–35	0.442	0.072	0.000	***	0.246	0.637
	> 55	< 26	0.726	0.083	0.000	***	0.499	0.954
		26–35	0.552	0.085	0.000	***	0.320	0.784
		36–45	0.291	0.093	0.015	*	0.038	0.544
**Risk Perception**	< 26	36–45	-0.221	0.046	0.000	***	-0.346	-0.096
		46–55	-0.326	0.051	0.000	***	-0.466	-0.187
		> 55	-0.333	0.061	0.000	***	-0.500	-0.166
	26–35	36–45	-0.147	0.047	0.016	*	-0.276	-0.018
		46–55	-0.252	0.052	0.000	***	-0.395	-0.109
		> 55	-0.258	0.062	0.000	***	-0.429	-0.088
	36–45	< 26	0.221	0.046	0.000	***	0.096	0.346
		26–35	0.147	0.047	0.016	*	0.018	0.276
	46–55	< 26	0.326	0.051	0.000	***	0.187	0.466
		26–35	0.252	0.052	0.000	***	0.109	0.395
	> 55	< 26	0.333	0.061	0.000	***	0.166	0.500
		26–35	0.258	0.062	0.000	***	0.088	0.429

*Notes:* *The mean difference is significant at the 0.05 level. **The mean difference is significant at the 0.01 level. ***The mean difference is significant at the 0.001 level.

**Table 4:** Post-Hoc (Tukey HSD) analysis – mean comparisons for self-reported traffic crashes suffered by cyclists (last 5 years). Factor: age group.

**Dependent variable**	**(I) Age group**	**(J) Age group**	**Mean Diff. (I-J)**	**Std. Error**	**Sig.**		**95% CI**	
					***p***	**Level**	**Lower**	**Upper**
**Traffic Crashes (5 Years)**	< 26	26–35	0.266	0.073	0.002	**	0.070	0.460
		36–45	0.442	0.090	0.000	***	0.200	0.690
		46–55	0.485	0.101	0.000	***	0.210	0.760
		> 55	0.603	0.121	0.000	***	0.270	0.930
	26–35	< 26	-0.266	0.073	0.002	**	-0.460	-0.070
		> 55	0.337	0.123	0.048	*	0.000	0.670
	36–45	< 26	-0.442	0.090	0.000	***	-0.690	-0.200
	46–55	< 26	-0.485	0.101	0.000	***	-0.760	-0.210
	> 55	< 26	-0.603	0.121	0.000	***	-0.930	-0.270
		26–35	-0.337	0.123	0.048	*	-0.670	0.000

*Notes:* *The mean difference is significant at the 0.05 level. **The mean difference is significant at the 0.01 level. ***The mean difference is significant at the 0.001 level.

In addition, the Supplementary SPSS dataset (.sav) will allow researchers to perform additional tests and comparisons using the entire set of measured variables.

## 2 Experimental design, materials, and methods

### 2.1 Participants

For this cross-sectional research, it was collected and analyzed the data of *n* = 1064 bicyclists (413 females, and 651 males) from 3 different territories: Latin America (*n =* 831 individuals, 78.1% of the sample; 38.6% women and 61.4% men), Europe (*n =* 161 individuals, 15.15% of the sample; 41% females and 59% males), and North America (*n =* 72 individuals, 6.75% of the sample; 37.5% women and 61.1% men).

In accordance with the pursued analyses and some previous research experiences dealing with different groups of cyclists divided by age [2], [3], considering it as a key variable for explaining road-related risks [4], [5], the full sample was divided in five intervals, composed as follows: < 26 years (*n =* 390, composing 36.7% of the sample); 26–35 years (*n =* 318, composing 29.9% of the sample); 36–45 years (*n =* 160, composing 15.1% of the sample); 46–55 years (*n =* 120, composing 11.2% of the sample); and > 55 years (*n =* 76, composing 7.1% of the sample).

### 2.2 Questionnaire

The questionnaire was administrated only in Spanish and consisted of four sections. The first part asked about individual and demographic variables, such as age, gender, region of provenance and main occupation.

In the second part, self-reported risky cycling behaviors were assessed using the raw item bank of the Cyclist Behavior Questionnaire (CBQ) [6], a self-report measure on road behavior specifically designed to measure high-risk riding behaviors (errors and violations) among bike users. This Likert scale is originally composed of 44 items distributed in three factors: *Violations* (*α =* 0.785), consisting of 16 items; *Errors* (*α =* 0.850), composed of 16 items; and *Positive Behaviors* (*α =* 0.729), consisting of 12 items, and based on the one developed by Özkan and Lajunen [7] for motor-vehicle drivers. The entire questionnaire used a frequency-based response scale of 5 levels: 0 = never; 1 = hardly ever; 2 = sometimes; 3 = frequently; 4 = almost always. A global score of *Risky Behaviors* (*α =* 0.895) was built up through the sum of Errors and Violations reported by respondents.

As for the third part, and in order to measure the *risk perception* and the *knowledge of traffic rules*, the Cyclist Risk Perception and Regulation Scale (RPRS) was used [8], Likert scale composed of 12 items: 7 for risk perception (*α =* 0.657), and 5 for assessing general rules of bike using (*α =* 0.722), in which the degree of perceived risk in objective risk factors and the knowledge of general regulations on the road are assessed in a scale from 0 (no knowledge/risk perceived) to 4 (highest knowledge/risk perceived).

Finally, the fourth part of the questionnaire consisted of a series of questions related to the use of bikes, such as the average use of the bicycle (including mean distances traveled and length of journeys) and the reasons for using it as a mean of transportation.

### 2.3 Statistical analysis

First of all, basic descriptive analyses (i.e. means and standard deviations of the study variables) were obtained, with the aim of establishing trends on aberrant (errors and violations) and positive cycling behaviors, protective factors such as the knowledge of traffic norms and risk perception, and their self-reported traffic crash rates as cyclists, based in their age groups - using five intervals, as described in the sample section. Finally, a set of comparative analyses (Tukey׳s Post-Hoc tests) were performed in order to determine significant differences between the specific age groups. Please note that the global score on risky behaviors is not included in the graphical presentation of the data, due to it constitutes the sum of two sub-scales (error and violation) of the CBQ. Nevertheless, the full set of variables is available in the annex dataset.
